# Differences in Plantar Pressure Distribution Between Adults with Asymptomatic and Symptomatic Flexible Flatfeet During Walking and Heel-Strike Running

**DOI:** 10.3390/s26082451

**Published:** 2026-04-16

**Authors:** Nicolas Haelewijn, Iris Deknudt, Marie Vanhaelewyn, Filip Staes, Evie Vereecke, Kevin Deschamps

**Affiliations:** 1 Musculoskeletal Rehabilitation Research Group, Department of Rehabilitation Sciences, KU Leuven, Spoorwegstraat 12, 8200 Brugge, Belgium; nicolas.haelewijn@kuleuven.be (N.H.); marie.vanhaelewyn@gmail.com (M.V.); 2 Musculoskeletal Rehabilitation Research Group, Department of Rehabilitation Sciences, KU Leuven, Tervuursevest 101, 3000 Leuven, Belgium; filip.staes@kuleuven.be; 3 Department of Development & Regeneration, Campus Kulak, KU Leuven, Etienne Sabbelaan 53, 8500 Kortrijk, Belgium; evie.vereecke@kuleuven.be

**Keywords:** flatfoot, asymptomatic, symptomatic, adults, flexible, plantar pressure

## Abstract

**Highlights:**

**What are the main findings?**

- Adults with symptomatic flexible flatfeet showed significantly increased plantar pressure in the midfoot and lateral forefoot regions during walking and heel-strike running.
- Symptomatic individuals adopted a lateralized pressure distribution pattern, contrasting the traditional expectation of medial overload in flatfoot conditions.

**What are the implications of the main findings?**

- Pain or instability in symptomatic flatfeet may lead to compensatory offloading of the medial arch, emphasizing the need for dynamic gait assessments over static posture evaluations.
- Plantar pressure analysis can inform more targeted rehabilitation strategies and prevent symptom progression in flexible flatfoot cases.

**Abstract:**

Flatfeet involve a collapse of the medial longitudinal arch, hindfoot valgus, and forefoot abduction. Flexible flatfoot is the most common type and can often be corrected with physiotherapy or orthotics. While some individuals remain asymptomatic, others develop symptoms for reasons that are not fully understood. This cross-sectional study compared plantar pressure distributions in 16 adults with asymptomatic and 16 with symptomatic flexible flatfeet (FPI-6 > 6; navicular drop > 5 mm), using a resistive-sensor-equipped pressure plate during walking and heel-strike running. During walking, symptomatic participants showed significantly higher total and peak forces at metatarsal 5 (*p* ≤ 0.003), and the midfoot (*p* ≤ 0.02146). The medial heel had significantly lower peak force (*p* = 0.00147), and metatarsal 4 showed higher peak force (*p* = 0.02539). Force ratios indicated a more lateralized pressure distribution in the symptomatic group. During heel-strike running, the symptomatic group exhibited higher total and peak forces at the fifth metatarsal, the midfoot, and the first metatarsal, with shorter time to peak force in the midfoot and the medial part of the heel. No significant ratio differences were found during running. Symptomatic individuals adopted a lateralized pressure distribution pattern, contrasting the traditional expectation of medial overload in flatfoot conditions.

## 1. Introduction

Foot posture is commonly classified as high-arched, neutral, or flatfoot based on the height of the medial longitudinal arch (MLA). Flatfoot posture may be unilateral or bilateral and is characterized by a collapse of the MLA, hindfoot valgus (calcaneal eversion), and forefoot abduction [1]. Deviations from neutral foot posture (e.g., excessive foot pronation and/or flat fleet) as a clear-cut cause of foot or lower-limb pathology remains poorly supported by evidence, and when associations do appear, they tend to be weak and inconsistent rather than strong causal links [2,3]. Flatfoot posture has specifically been linked to an increased risk of medial tibial stress syndrome [4], plantar fasciopathy [5], patellofemoral pain [6], and low back pain [7]. The prevalence of flatfoot is estimated at approximately 20% in the general population, with higher rates reported in younger individuals and females [8,9]. Flatfoot is further classified as flexible or rigid. Flexible flatfoot presents with a normal MLA in non-weight-bearing conditions which collapses under load [10]. In contrast, rigid flatfoot is characterized by a persistently collapsed MLA in both non-weight-bearing and weight-bearing conditions [10], typically more associated with structural pathology (e.g., tarsal coalition or arthritis) [11].

Not all individuals with flexible flatfeet are symptomatic. Although common as an anatomical variant, the mechanisms underlying symptom development remain unclear. Structural studies have yielded mixed findings. For example, Moraleda & Mubarak (2011) reported no significant differences in forefoot, midfoot, or hindfoot alignment between symptomatic and asymptomatic individuals, but identified a greater anteroposterior talonavicular coverage angle in symptomatic cases, indicating increased lateral navicular displacement [12]. Other studies have highlighted alterations in kinematic patterns. Zhang et al. (2019) demonstrated altered kinematics in symptomatic individuals, including increased peak forefoot abduction during gait, suggesting impaired control of foot motion [13]. However, these findings rely on advanced imaging and motion analysis, limiting their clinical applicability.

Plantar pressure measurement provides a more direct assessment of loading and force transmission during gait. During walking, flatfeet demonstrate distinct plantar pressure characteristics [14,15], findings that have been corroborated by other studies [6,16]. Collectively, these results indicate that plantar pressure analysis can characterize foot-posture-related loading patterns. In their systematic review, Buldt & Allan et al. (2018) synthesized evidence from sixteen studies examining the association between foot posture and plantar pressure during gait [14]. Individuals with flatfeet consistently exhibited higher peak pressure, pressure–time integral, maximum force, force–time integral, and contact area in the medial arch, central forefoot, and hallux, alongside lower values in the lateral and medial forefoot. Additionally, the center of pressure trajectory was more medially deviated in planus feet [14].

However, interpretation of these findings is complicated by inconsistencies in flatfoot definition and diagnostic criteria, as well as the frequent omission of distinctions between symptomatic and asymptomatic individuals. Furthermore, most studies have focused exclusively on walking. To date, no studies have incorporated more demanding functional tasks such as running. Therefore, the aim of this study is to compare plantar pressure distribution during walking and heel-strike running between individuals with asymptomatic and symptomatic flexible flatfeet. We hypothesize that symptomatic individuals will demonstrate higher peak forces at the midfoot and forefoot regions. Furthermore, we expect altered force ratio patterns throughout stance, including reduced rearfoot loading and increased midfoot and forefoot loading during mid- and terminal stance.

## 2. Materials and Methods

The aim of this cross-sectional study was to investigate the differences in multi-segment foot joint plantar pressure distributions between adults with asymptomatic and symptomatic flexible flatfeet during walking and heel-strike running. The data was collected at the We-lab of the KU Leuven Bruges Campus.

### 2.1. Participant Details

Participants signed a written consent form prior to participation in accordance with the Declaration of Helsinki and a written ICF (International Classification of Functioning, Disability and Health) form was obtained. The local ethics committee (Commissie Medische Ethiek UZ/K.U. Leuven S67036) approved the study. The asymptomatic participants were recruited via advertisements at the KU Leuven Bruges Campus consisting of flyers and posts on social media. Participants in the symptomatic flexible flatfoot group were diagnosed by a physician from the local hospitals: AZ Sint Lucas or AZ Sint Jan. Symptoms were defined by the presence of one or more of the following: foot pain rated above four on a 0–10 Visual Analog Scale (VAS), medial arch pain, metatarsalgia (including general or diffuse hyperkeratosis), lateral impingement pain, sinus tarsi pain or tibialis posterior dysfunction in stages I and II (without evidence of rupture), accompanied by a painful single heel raise test. Furthermore, participants were adults between 18 and 65 years old, who were able to run at a self-selected low speed and were a minimum of one hour and a half to a maximum of six hours physically active per week. All participants were required to have an FPI-6 greater than six and a navicular drop test result greater than 5 mm. Subjects aged below 18 or above 65 were excluded from this study (Table 1).

Further exclusion criteria were: any medical contraindication for physical activity, systemic disorders, recent surgical interventions at the lower limb (<6 months), leg length difference >3 cm, pregnancy, BMI > 30 kg/m^2^, chronic ankle pain, ankle fractures, single hyperkeratotic lesion with nucleus under the metatarsal head, recent participation in a rehabilitation program (<3 months), or recent orthotic therapy (<3 months). Upon completion of recruitment, a total of 32 participants were selected and divided into two groups: an asymptomatic (*n* = 16) and a symptomatic (*n* = 16) group (Table 1). The two groups were matched for age, sex, and body mass index (BMI) to minimize the potential influence of these confounding factors on plantar pressure outcomes, as these variables are known to affect biomechanical and anthropometric characteristics.

### 2.2. Data Collection and Instrumentation

The dynamic barefoot plantar pressures of the dominant foot were measured with individuals walking at a self-selected speed until five ‘representative’ walking and running trials were recorded in each condition. Participants walked or ran only in one direction. A trial was considered representative if the participants made clear pedobarographic contact with good inter-trial consistency, judged by the visual inspection of an experienced researcher. One experienced clinician performed all the gait analyses. The laboratory consisted of a 16.5 m long walkway integrating an AMTI force plate (model: ZBP 460 × 2070-6-1000; Advanced Mechanical Technology Inc., Watertown, MA, USA), which is seen as the gold standard [17], with a Footscan^®^ plantar pressure plate (Materialise Motion, Paal, Belgium, 0.5 m × 0.4 m × 0.12 m, with 4096 resistive sensors, 200 Hz, 4 sensors/cm^2^) on top [18]. The temporal synchronization of both the force plate and pressure plate was performed with a 3D Box© (Materialise Motion, Paal, Belgium). This combined setup was used to exploit the complementary strengths of both systems. The pressure plate provided detailed spatial information on plantar load distribution, which was required for regional analyses, whereas the force plate provided an accurate measurement of the vertical ground reaction force. The latter was used to verify and, where necessary, scale the total force signal derived from the pressure plate, thereby improving the accuracy and reliability of force–time variables calculated from the plantar pressure data.

Walking and running speed were measured using a motion capture approach in which a reflective marker was placed on the subject’s sacrum to track pelvic movement. The tracking of the marker was performed with a passive optoelectronic system (Vicon Motion System, Ltd., Oxford Metrics, Oxford, UK) equipped with 12 infrared cameras (Vero, Vicon Motion Systems, Ltd., Oxford Metrics, Oxford, UK).

### 2.3. Plantar Pressure Mapping Method

**Figure 1:**
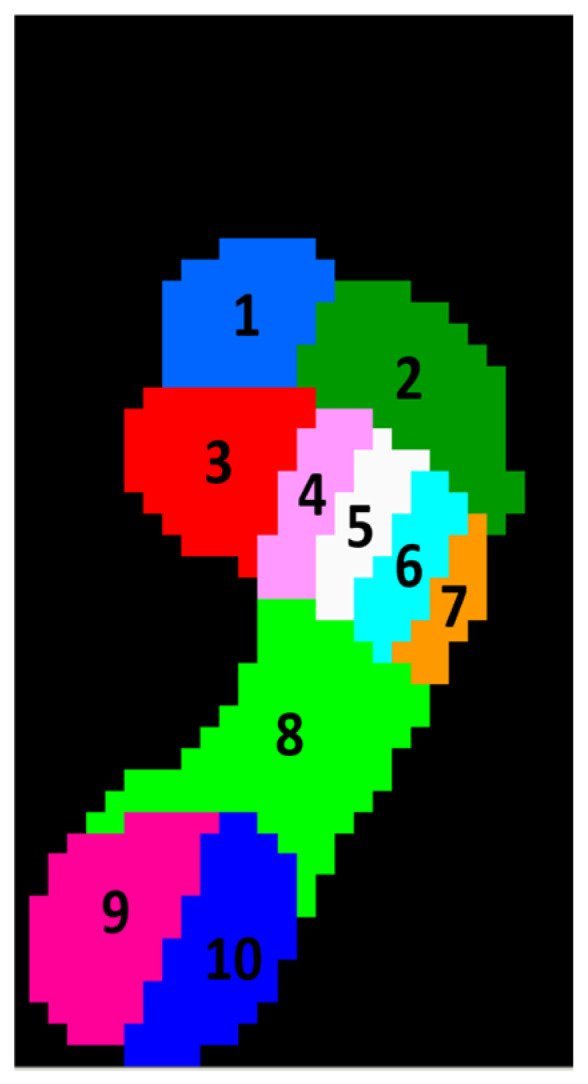
Semi-automatic total mapping method used for segmenting the foot into the different regions of interest: (1) hallux (T1), (2) toes 2–5 (T2-5), (3) first metatarsal, (4) second metatarsal, (5) third metatarsal, (6) fourth metatarsal, (7) fifth metatarsal, (8) midfoot, (9) medial heel (HM), (10) lateral heel (HL).

The plantar pressure measurements were analyzed using Footscan^®^ 7.92 gait 2nd generation software (Materialise Motion, Paal, Belgium). This program integrates a semi-automatic total mapping method [19] allowing the segmentation of the total pedobarographic field in a supervised way by the authors. This segmentation consisted of several steps. First, the foot axis was adjusted to align with metatarsal II and mid heel. Next, the anteroposterior subdivision was performed by dividing the foot into four regions of interest: toes, forefoot, midfoot and hindfoot. Subsequently, a medio-lateral division of the forefoot region was performed, separating the five individual metatarsals. Furthermore, the heel area was divided in two, a medial and a lateral heel region [19]. As a result, this method defines ten regions of interest on the peak pressure footprint (Figure 1): hallux (T1), toes two to five (T2-5) considered as one region, the individual metatarsal heads one to five (MTH1-5), midfoot (MF), medial heel (HM) and lateral heel (HL).

For each region of the ten regions of interest the peak force and total force were extracted. Peak force was defined as the highest value recorded by any plantar pressure sensor within a region, and total force as the sum of all sensor values in that region. To better characterize temporal loading differences between segments, several force indices were analyzed. An extensive description of these indices can be found elsewhere [19]. The ratio of first to fifth metatarsal (MTH1/MTH5) loading was used to quantify medial–lateral load distribution in the forefoot. The hallux-to-first metatarsal ratio (HALLUX/MTH1) was considered to capture force distribution within the first ray. The medial–lateral force distribution of the entire foot (MEDIAL/LATERAL) quantified loading differences between the medial and lateral sides. The rearfoot-to-forefoot ratio (REARFOOT/FOREFOOT) indicated force distribution between the hindfoot (medial and lateral heel) and the forefoot (five metatarsal heads), while the medial–lateral forefoot ratio (MEDIAL FOREFOOT/LATERAL FOREFOOT) examined relative load across the forefoot. Finally, the rearfoot-to-midfoot ratio (REARFOOT/MIDFOOT) assessed force transfer between the rearfoot and midfoot. These temporal indices were interpolated to 100 points representing the stance phase of the gait cycle (0–100%), with 0% corresponding to initial heel contact and 100% to the push off phase.

### 2.4. Statistical Analysis

The peak force and total force data of both groups were compared using two-tailed paired t-tests since the groups were matched for age, sex, and BMI. The temporal loading indices were statistically compared using one-dimensional statistical parametric mapping (SPM) (SPM1D version M.0.4.10, www.spm1d.org) analyses, which involved conducting paired t-tests at each time point of the gait cycle. SPM was used to analyze time-normalized landing phase profiles. This approach allows for a more comprehensive assessment of temporal variations while avoiding statistical issues related to multiple comparisons across time-series data. SPM achieves this by calculating a test statistic profile across each time point and modeling the behavior of random time-dependent signals with a similar smoothness to the recorded data [20].

## 3. Results

### 3.1. Walking

#### 3.1.1. Peak and Total Force

The symptomatic group showed a significantly higher total force under MTH5 (*p* < 0.001) and in the MF (*p* = 0.0215) and a lower total force under the HM segment (*p* = 0.0147). Regarding the peak force, the symptomatic group showed significantly higher forces under MTH 5 (*p* = 0.003), MTH 4 (*p* = 0.025) and the MF (*p* = 0.0036) (Table 2).

#### 3.1.2. Force Ratio

**Figure 2:**
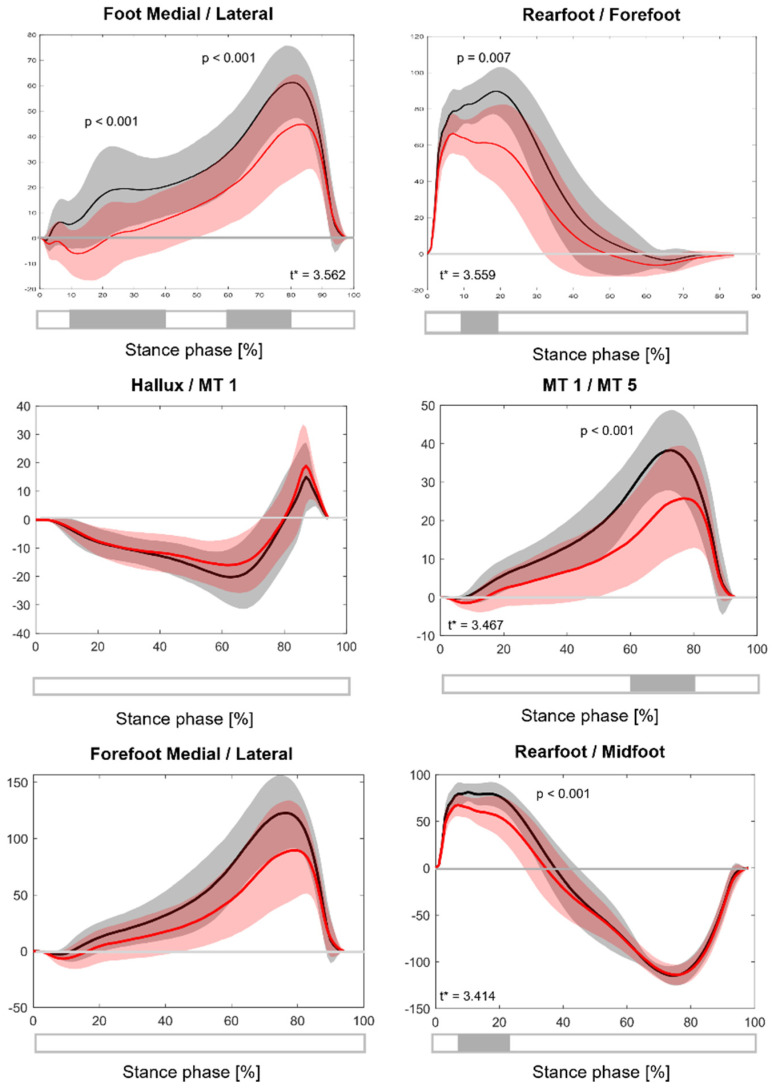
Normalized force ratios between foot segments during the stance phase of walking. The black line indicates the asymptomatic flatfoot group; the red line, the symptomatic foot. *: statistical significance. Shaded areas represent standard deviation band. *Y*-axis: unitless indices [17].

During the stance phase of gait, the symptomatic group displayed notable differences in different force ratios (Figure 2). The medial/lateral ratio indicated a shift toward increased lateral and reduced medial loading, particularly between 10 and 40% (*p* < 0.001) and 60 and 80% (*p* < 0.001) of the stance phase. The MTH1/MTH5 ratio further demonstrated reduced loading on the first metatarsal (MTH1) and increased loading on the fifth metatarsal (MTH5) between 60 and 80% of the stance phase (*p* < 0.001). In the rearfoot/forefoot ratio, the symptomatic foot exhibited decreased rearfoot loading and increased forefoot loading during early stance (approximately 0–30%) (*p* = 0.007). Finally, the rearfoot/midfoot ratio showed significantly greater midfoot loading relative to the rearfoot between 10 and 20% of the stance phase (*p* < 0.001).

### 3.2. Heel-Strike Running

#### 3.2.1. Peak and Total Force

The symptomatic group showed a higher total force under the MTH 5 (*p* < 0.0001) and the MF (*p* = 0.016) during heel-strike running. We also observed significantly higher peak forces under the MTH 1 (*p* = 0.034), MTH 5 (*p* = 0.002) and the MF (*p* = 0.0015) in the symptomatic group during running (Table 3).

#### 3.2.2. Force Ratio

No significant data were found in plantar force distribution during heel-strike running.

## 4. Discussion

This study aimed to examine differences in plantar pressure distribution between adults with asymptomatic and symptomatic flexible flatfeet during level walking and heel-strike running. During walking, the symptomatic group exhibited significantly greater total and peak forces in the midfoot. Peak forces were also significantly elevated in the fourth and fifth metatarsal regions (M4 and M5), and time to peak force was significantly shorter in M4, indicating a more rapid loading rate in the lateral forefoot. In this context, the lateral metatarsals may assume a greater propulsive role, compensating for the underactive medial forefoot. The aforementioned pattern can be described as a “low gear push off” pattern [21]. In normal gait, an efficient “high gear push off” primarily occurs through the first and second metatarsals, which are aligned with the medial forefoot and supported by the Windlass mechanism and first MTP joint dorsiflexion [21]. This mechanism optimizes propulsion, elastic recoil, and efficient energy transfer. From a clinical perspective, the present findings may have implications for orthotic design and rehabilitation. If symptomatic flexible flatfoot is associated with a more laterally directed push off pattern, interventions may need to address not only medial arch support but also forefoot load progression during the propulsion phase. In that context, orthotic strategies aimed at improving medial column support and facilitating a more efficient transition of load across the forefoot may be relevant, rather than focusing exclusively on presumed medial overload. Similarly, rehabilitation may benefit from targeting functional deficits that could contribute to altered propulsive mechanics, such as impaired foot intrinsic and extrinsic muscle performance. These clinical implications should be interpreted with caution, as plantar pressure data alone do not establish the specific kinematic or neuromuscular mechanisms underlying the observed loading pattern.

In contrast, peak force in the medial heel was significantly reduced in the symptomatic group, potentially reflecting decreased reliance on the rearfoot as a primary shock-attenuating structure [22]. Collectively, these findings may suggest an altered load transfer pattern characterized by reduced rearfoot contribution and increased midfoot and forefoot loading. The statistical parametric mapping of force ratios across the stance phase provided further insight into the temporal characteristics of loading. The symptomatic group demonstrated a more lateralized pressure distribution, with relatively greater loading of the fifth metatarsal and reduced loading of the first metatarsal from midstance to push off. The medial–lateral force ratio confirmed significantly reduced medial and increased lateral loading between 10 and 40% and 60 and 80% of stance. In addition, symptomatic individuals exhibited reduced rearfoot loading and increased forefoot loading during early stance, as well as greater midfoot loading relative to the rearfoot between 10 and 20% of stance. These findings indicate that symptomatic flexible flatfoot may be associated with a compensatory redistribution of plantar pressure toward the lateral column.

Traditionally, flatfoot posture has been associated with excessive medial loading due to MLA collapse and overpronation [14,23]. However, the present findings suggest that symptomatic individuals may exhibit an opposing dynamic pattern. This observation is consistent with the work of Zhang et al. (2019), who reported impaired medial control in symptomatic flatfoot [13]. Reduced medial stability may limit effective load acceptance along the medial column, thereby promoting lateral load transfer as a compensatory mechanism. Additionally, Blackwood et al. report differences in all variables with an emphasis in the medial midfoot [24]. Moreover, the combination of reduced rearfoot loading and increased midfoot loading could suggest alterations in sagittal plane load progression. Insufficient structural and muscular support of the MLA may indicate a compromised controlled shock absorption during early stance, thereby modifying proximal–distal load transmission.

During heel-strike running, the symptomatic group demonstrated significantly greater peak forces in M5, the midfoot, and M1, along with a significantly shorter time to peak force in the medial midfoot, suggesting a more rapid and potentially less controlled loading response. In contrast to walking, no significant differences in force ratio trajectories were observed during running. This absence of temporally distinct group differences may be attributable to greater inter-individual variability in running mechanics [25] or to converging toward similar loading strategies at higher gait speeds to enhance mechanical efficiency and reduce injury risk. Kirmizi et al. (2020) similarly reported that plantar pressure characteristics may become less distinguishable between foot types as velocity increases [26]. Nevertheless, the elevated regional peak forces observed in the symptomatic group may indicate that altered plantar loading persists under higher mechanical demands, particularly in the midfoot and lateral forefoot, which may reflect compromised MLA stability during dynamic propulsion.

Overall, the hypotheses were only partially supported. Although increased midfoot peak forces and a shorter time to peak force were observed in symptomatic individuals, especially during walking, the anticipated increase in medial forefoot and hallux loading was not confirmed. Instead, a predominantly lateralized pressure distribution was identified in the symptomatic group. This finding challenges the conventional assumption that symptomatic flatfoot is characterized primarily by medial overload secondary to arch collapse. Rather, symptom manifestation may be more closely related to altered dynamic control and the compensatory lateral redistribution of load. These findings have important implications for clinical assessment and intervention. Recent research by Mahmoudiyan et al. [27] found that insoles led to increased peak plantar pressure in the medial midfoot. This systematic review further strengthens our findings that the lateralized loading pattern observed in symptomatic individuals indicates that rehabilitation should not solely focus on reducing medial overload. Furthermore, our results highlight the complementary value of dynamic plantar pressure assessment. While static measures such as the six-item foot posture index provide information regarding structural alignment, they do not capture functional load redistribution during movement. Pressure-sensing technologies, as applied in the present study, offer an objective, time-resolved quantification of plantar loading and may therefore enhance the detection of compensatory strategies associated with symptom development.

Several limitations should be considered. Plantar pressure data were obtained using a pressure plate system, which does not provide information on joint kinematics or muscle activation patterns. Consequently, the biomechanical mechanisms underlying the observed loading differences, including segmental motion and neuromuscular control strategies, could not be directly assessed. The sample size was relatively modest, which may have limited statistical power to detect more subtle differences, particularly during running. Future studies with larger cohorts may benefit from regression analyses to identify key plantar pressure parameters predictive of flatfeet. Furthermore, the symptomatic participants tended to be older than the asymptomatic participants. This age trend should be considered when interpreting the findings, as age-related changes in tissue elasticity, muscle strength, and joint mobility may influence plantar loading patterns. In addition, clinical heterogeneity within the symptomatic group may have contributed to variability in plantar loading outcomes, particularly in peak pressure measures. The symptomatic cohort included individuals presenting with different foot-level complaints (e.g., medial arch pain, metatarsalgia, or lateral impingement), which may reflect partially distinct compensatory mechanisms. Variability in participant characteristics is a recognized source of heterogeneity in clinical research and can influence the interpretation of observed effects. Although the present study restricted inclusion to symptoms related to flexible flatfoot—thereby maintaining a relatively specific clinical phenotype compared to broader definitions used in parts of the literature—differences in symptom presentation within the foot may still have affected the results. Future studies may benefit from investigating more homogeneous subgroups or exploring subgroup-specific loading patterns, where sample size and recruitment feasibility allow. At the same time, future studies may benefit from examining more specific symptomatic subgroups, where feasible, to determine whether distinct clinical presentations are associated with different plantar pressure adaptations. In addition, only walking and heel-strike running were examined, which may not fully represent the range of the functional demands encountered in daily activities. Future investigations should incorporate larger cohorts, multimodal biomechanical assessment, and a broader range of dynamic tasks. Longitudinal designs may further clarify whether specific plantar pressure profiles are predictive of symptom development in individuals with flexible flatfeet. Finally, no neutral-foot control group was included. A control group can provide a useful baseline for contextualizing findings relative to typical gait patterns; however, the present study was specifically designed to compare symptomatic and asymptomatic individuals within the flexible flatfoot spectrum, in order to better isolate characteristics associated with symptom presence under a similar structural phenotype. Therefore, the focus was placed on intra-condition variability rather than comparisons with normative gait. Nevertheless, the absence of a neutral foot posture index (FPI) group limits the ability to determine whether the observed plantar pressure patterns represent deviations specific to symptomatic flatfoot or differences relative to the general population. Future studies incorporating a neutral FPI control group would help further contextualize these findings.

## 5. Conclusions

In conclusion, adults with symptomatic flexible flatfeet display altered plantar pressure patterns compared with asymptomatic individuals, notably greater midfoot and lateral forefoot loading and reduced rearfoot contribution during walking. These findings reflect a compensatory lateral load redistribution and a “low gear push off” pattern rather than the traditionally assumed medial overload. Elevated peak forces and rapid loading responses during running indicate that altered plantar loading persists under higher dynamic demand, despite less distinct temporal differences. This suggests compromised medial column control and altered load progression could be associated with symptom manifestation. The dynamic plantar pressure assessment adds functional insight beyond static structural measures and may better detect compensatory strategies. Clinically, interventions should target dynamic medial stability and intrinsic foot muscle function in addition to addressing medial loading. Future research with larger samples and multimodal biomechanical analysis is needed to clarify the mechanisms and predictive value of these pressure profiles.

## Figures and Tables

**Table 1:** Demographic data and clinical data of both groups.

	Asymptomatic Flatfeet (Mean ± SD)	Symptomatic Flatfeet (Mean ± SD)	Paired *t*-Test (*p*)
Height (m)	1.75 ± 0.09	1.74 ± 0.09	0.78
Weight (kg)	74 ± 11.74	80.81 ± 15.82	0.18
BMI (kg/m^2^)	24.22 ± 3.46	26.65 ± 4.49	0.1
Shoe size (EU size)	41.75 ± 2.91	42.06 ± 2.82	0.76
Age (years)	34.19 ± 13.51	43.50 ± 15.40	0.08
Walking speed (m/s)	1.3 ± 0.1	1.2 ± 0.1	0.12
Running speed (m/s)	2.6 ± 0.4	2.4 ± 0.3	0.22
FPI-6	8.5 ± 2.0	9.31 ± 2.12	0.27
Navicular drop (mm)	8.31 ± 3.4	8.13 ± 3.10	0.87

**Table 2:** Mean and SD for the total force and peak forces observed in both study cohorts in the walking condition (* denotes *p* < 0.05).

		Asymptomatic Flatfeet (Mean ± SD)	Symptomatic Flatfeet (Mean ± SD)	Paired Samples *t*-Test (*p*)
Total Force (N)				
	Hallux	184.6 ± 57.9	182.5 ± 58.8	0.93
	Toe 2–5	55.1 ± 29.3	65.7 ± 37.2	0.49
	MTH 1	248.1 ± 75.2	216 ± 58.5	0.12
	MTH 2	201.5 ± 45.9	204.8 ± 62.9	0.86
	MTH 3	153 ± 40.2	153 ± 40.1	0.99
	MTH 4	89.4 ± 37.4	112.4 ± 28.2	0.053
	MTH 5	30.6 ± 15.8	83.7 ± 38.1	>0.01 *
	Midfoot	97.2 ± 66.2	176.9 ± 109	>0.01 *
	Heel Medial	345 ± 87	264.9 ± 58.8	>0.01 *
	Heel Lateral	267.4 ± 56	270.7 ± 54.2	0.86
Peak Force (N)				
	Hallux	15.6 ± 3.9	16.2 ± 5.2	0.71
	Toe 2–5	5.4 ± 3	6.5 ± 2.9	0.4
	MTH 1	12.4 ± 2.8	12.7 ± 2.8	0.77
	MTH 2	14.8 ± 1.7	15 ± 3.3	0.87
	MTH 3	12.8 ± 1.5	14 ± 3.6	0.3
	MTH 4	8.6 ± 2.5	11.3 ± 2.8	0.03 *
	MTH 5	5.0 ± 1.8	8.9 ± 3	>0.01 *
	Midfoot	3.4 ± 1.8	6.5 ± 2.4	>0.01 *
	Heel Medial	14.6 ± 1.7	15.3 ± 4.1	0.51
	Heel Lateral	13.8 ± 1.7	15.1 ± 4.1	0.25

**Table 3:** Mean and SD for the total force and peak forces observed in both study cohorts in the running condition (* denotes *p* < 0.05).

		Asymptomatic Flatfeet (Mean ± SD)	Symptomatic Flatfeet (Mean ± SD)	Paired Samples *t*-Test (*p*)
Total Force (N)				
	Hallux	228.5 ± 73.6	206.6 ± 51.9	0.36
	Toe 2–5	85.6 ± 27.4	97.8 ± 59.5	0.44
	MTH 1	425.8 ± 105.6	402.8 ± 136.8	0.54
	MTH 2	249.7 ± 59.5	284.3 ± 69.5	0.14
	MTH 3	198.3 ± 63.7	182.4 ± 44.6	0.47
	MTH 4	133 ± 45.1	152 ± 41	0.19
	MTH 5	41.7 ± 19	117.2 ± 45.3	>0.01 *
	Midfoot	240.2 ± 147.7	394.4 ± 215.9	0.02 *
	Heel Medial	404.4 ± 126.2	346.5 ± 140.7	0.23
	Heel Lateral	365.8 ± 105.5	332.4 ± 110	0.48
Peak Force (N)				
	Hallux	19.5 ± 5.5	19.1 ± 5.4	0.86
	Toe 2–5	7.9 ± 3.7	7.9 ± 3.6	0.97
	MTH 1	16.1 ± 3.7	20.0 ± 5.5	0.03 *
	MTH 2	14.9 ± 3.2	17.4 ± 4.7	0.76
	MTH 3	12.5 ± 2.5	14.7 ± 3.3	0.11
	MTH 4	9.4 ± 4.1	11.9 ± 3.1	0.79
	MTH 5	6.3 ± 3.9	11.1 ± 3.8	>0.01 *
	Midfoot	5.9 ± 2.7	8.6 ± 2.4	>0.01 *
	Heel Medial	19.2 ± 5.4	18.4 ± 8	0.77
	Heel Lateral	18.4 ± 5.3	17.8 ± 7.7	0.83

## Data Availability

The original contributions presented in this study are included in the article. Further inquiries can be directed to the corresponding author.
